# The Role of Extracellular Vesicles as a Shared Disease Mechanism Contributing to Multimorbidity in Patients With COPD

**DOI:** 10.3389/fimmu.2021.754004

**Published:** 2021-12-02

**Authors:** Laura V. Reid, C. Mirella Spalluto, Alastair Watson, Karl J. Staples, Tom M. A. Wilkinson

**Affiliations:** ^1^ Clinical and Experimental Sciences, Faculty of Medicine, University of Southampton, Southampton, United Kingdom; ^2^ National Institute for Health Research Southampton Biomedical Research Centre, Southampton Centre for Biomedical Research, Southampton General Hospital, Southampton, United Kingdom; ^3^ Birmingham Medical School, University of Birmingham, Birmingham, United Kingdom

**Keywords:** EV - extracellular vesicle, miRNA - microRNA, COPD - chronic obstructive pulmonary disease, multimorbidity, inflammation

## Abstract

Chronic obstructive pulmonary disease (COPD) is one of the leading causes of death worldwide. Individuals with COPD typically experience a progressive, debilitating decline in lung function as well as systemic manifestations of the disease. Multimorbidity, is common in COPD patients and increases the risk of hospitalisation and mortality. Central to the genesis of multimorbidity in COPD patients is a self-perpetuating, abnormal immune and inflammatory response driven by factors including ageing, pollutant inhalation (including smoking) and infection. As many patients with COPD have multiple concurrent chronic conditions, which require an integrative management approach, there is a need to greater understand the shared disease mechanisms contributing to multimorbidity. The intercellular transfer of extracellular vesicles (EVs) has recently been proposed as an important method of local and distal cell-to-cell communication mediating both homeostatic and pathological conditions. EVs have been identified in many biological fluids and provide a stable capsule for the transfer of cargo including proteins, lipids and nucleic acids. Of these cargo, microRNAs (miRNAs), which are short 17-24 nucleotide non-coding RNA molecules, have been amongst the most extensively studied. There is evidence to support that miRNA are selectively packaged into EVs and can regulate recipient cell gene expression including major pathways involved in inflammation, apoptosis and fibrosis. Furthermore changes in EV cargo including miRNA have been reported in many chronic diseases and in response to risk factors including respiratory infections, noxious stimuli and ageing. In this review, we discuss the potential of EVs and EV-associated miRNA to modulate shared pathological processes in chronic diseases. Further delineating these may lead to the identification of novel biomarkers and therapeutic targets for patients with COPD and multimorbidities.

## Introduction

Chronic obstructive pulmonary disease (COPD) is the third leading cause of death worldwide due to its prevalence, severity and the absence of an effective treatment to reverse disease progression (1). Individuals with COPD experience progressive lung function decline with periods of acute exacerbation (AECOPD) that impact quality of life and present a high economic burden due to direct medical costs and loss of working days (2–5). The development of more sensitive diagnostics and personalised treatments for COPD remains challenging due to our limited understanding of the complex molecular mechanisms underlying the disease (6).

The main pathological driver of COPD is inhalation of noxious stimuli such as cigarette smoke (CS) and particulate matter (PM) air pollution. Repeated exposure to respiratory toxins compromises the function of the immune system, induces chronic inflammation and directly damages structural cells, leading to emphysema and vascular remodelling (7, 8). In addition, damage caused by noxious stimuli promotes features of accelerated ageing including a state of cell cycle arrest, known as cellular senescence, that increases the release of inflammatory factors and has been associated with COPD pathogenesis (9). Furthermore exposure to noxious stimuli and age-associated immune alterations contribute to a dysfunctional immune response and increased susceptibility to acute respiratory infections observed in COPD (10–12). Respiratory infections result in elevation of airway and systemic inflammation on top of the chronic inflammation present in stable COPD and are the predominant cause of AECOPD (13).

**Figure 1:**
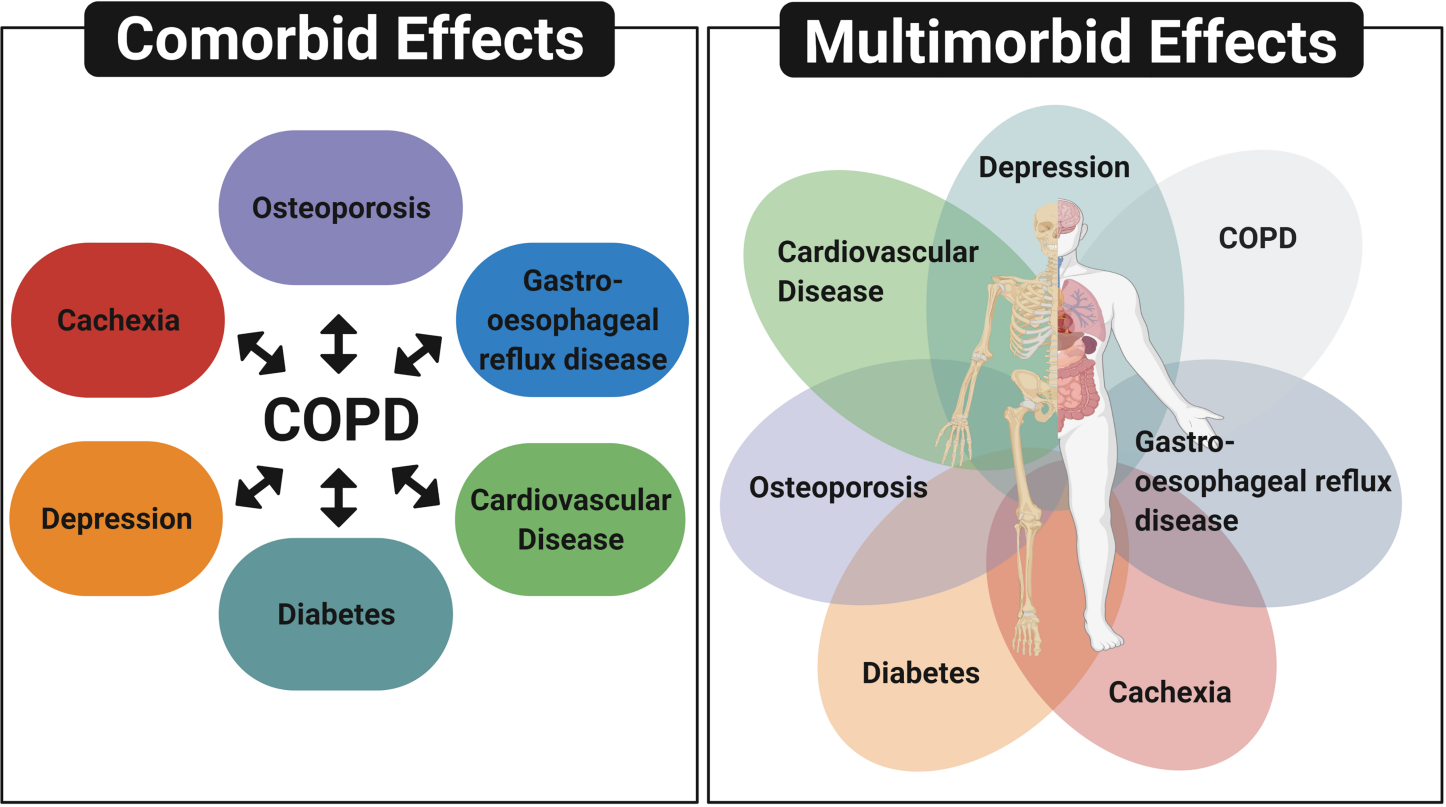
Comorbidity and multimorbidity in patients with COPD. The term ‘‘comorbidity’’ refers to the combined effects of additional conditions in reference to COPD, whereas “multimorbidity” indicates that no single condition holds priority over any of the co-occurring conditions. Diagram depicts other conditions commonly observed in patients with COPD, which include cardiovascular disease, depression, cachexia, diabetes, osteoporosis and gastro-oesophageal reflux disease. Created with BioRender.com.

The occurrence of multiple chronic conditions termed “comorbidities” or “multimorbidities” are common in patients with COPD (14, 15) (**Figure 1**). Other conditions commonly observed in conjunction with COPD include cardiovascular disease (CVD), diabetes, osteoporosis and gastro-oesophageal reflux disease (15). While the term ‘‘comorbidity’’ refers to the combined effects of additional conditions in reference to an index chronic condition, “multimorbidity” indicates that no single condition holds priority over any of the co-occurring conditions (16). As many patients with COPD have multiple concurrent chronic conditions, which require an integrative management approach, there is a need to greater understand the shared disease mechanisms contributing to multimorbidity.

Our current understanding of the mechanistic drivers for multimorbidity in COPD, including the role of shared risk factors were recently reviewed in detail by Burke and Wilkinson (15). These authors suggested that disease co-occurrence may not be by chance but as a result of shared genetic predisposition and responses to biological and environmental stressors (15). They also highlighted current and novel management strategies that target these underlying mechanisms. In support of this suggestion, network analyses have identified shared genes, proteins and biological pathways common to COPD and its most prevalent coexisting diseases (17). In addition, there is accumulating evidence that exposure to shared risk factors including inhalation of noxious stimuli and accelerated ageing may act as a central mechanism contributing to the development of multiple chronic diseases (18, 19). Furthermore, Burke and Wilkinson introduced the concept that extracellular vesicles (EVs) may contribute to dissemination of inflammation and therefore multimorbidity in COPD patients (15).

Recently EVs have been reported as local and systemic immune and inflammatory mediators that may act to spread or alleviate disease (15, 20–22). During acute and chronic inflammation, vascular permeability is dramatically increased and the alveolar-capillary barrier is reduced allowing lung inflammatory mediators such as proteins and EVs to reach the systemic circulation and potentially distant organs (21, 23). In this review we appraise the latest evidence around the potential role of EVs as circulating inflammatory mediators which could propagate systemic inflammation and multimorbidity in response to shared risk factors including ageing, inhaled noxious stimuli and respiratory infections.

## Extracellular Vesicles

**Figure 2:**
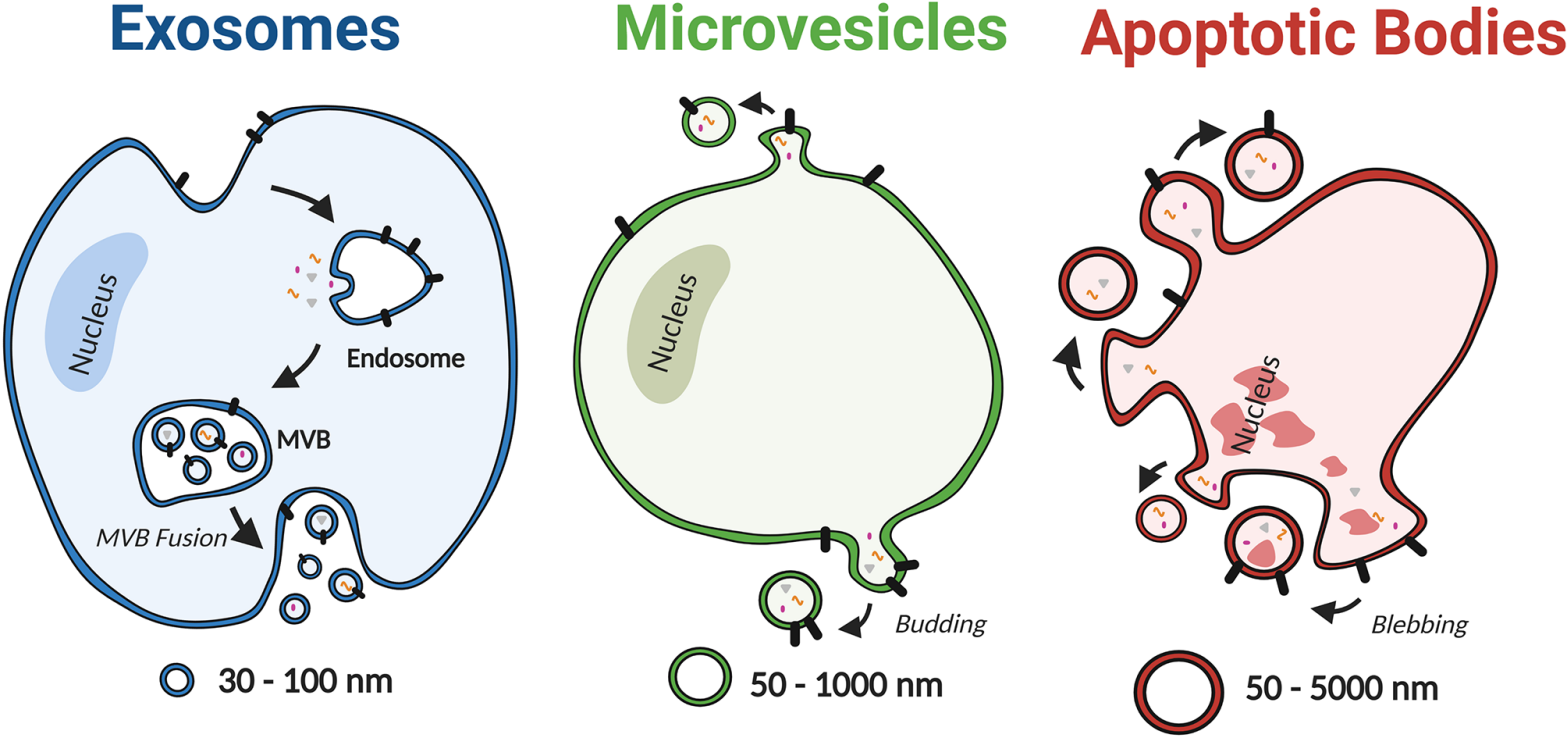
Schematic depiction of EV subtypes, including exosomes, microvesicles, and apoptotic bodies. Based on the mechanism of biogenesis, EVs can be categorised into three distinct subgroups; exosomes, microvesicles and apoptotic bodies. Exosomes are the smallest subclass of EVs with a diameter ranging from 30 nm to 100 nm. They are produced as intraluminal vesicles by inward budding of the endosomal membrane to form multivesicular bodies (MVB), which release vesicles upon fusion with the plasma membrane. In contrast, MVs are 50 nm to 1000 nm in diameter and are formed by direct budding from the plasma membrane. Lastly apoptotic bodies have the broadest range of diameters (50–5000 nm) and are produced by cells undergoing apoptosis. Created with BioRender.com.

EVs are highly heterogeneous lipid bilayer particles that have been isolated from a variety of cell types and biological fluids including serum, plasma, urine and bronchoalveolar lavage fluid (BALF) (24–30). They are generated and released by cells *via* a range of mechanisms that have been used to categorise EVs into three distinct subgroups; exosomes, microvesicles and apoptotic bodies (**Figure 2**) (31). However, our understanding of the role of specific EV subgroups remains limited due to the technical challenges associated with isolating pure subgroups, including EV size overlap and the current lack of subgroup specific markers (32). Therefore, this review will consider the overall role of EVs rather than specific subgroups.

EVs were originally considered to be cell debris but have since been shown to transfer lipids, proteins and nucleic acids locally and systemically as a form of intercellular communication mediating both homeostatic and pathological conditions (33, 34). Furthermore distinct EV-associated cargo have been reported depending on the origin and the physiological conditions including disease status (35, 36). EVs are enriched in surface proteins with immunoregulatory functions, such as the major histocompatibility molecules (MHC) class I and II (37). In addition, the transfer of immune and inflammatory mediators such as cytokines, chemokines and proteolytic enzymes *via* EVs has been shown to be altered in response to stimuli and to modulate recipient cell behaviour (38).

One of the most widely studied EV cargo are microRNAs (miRNAs). MiRNAs are short 17-24 nucleotide, non-coding RNA molecules that regulate gene expression by translational repression or degradation of mRNA (39). The miRNA content of EVs has been reported to be markedly different from that of the parent cell, suggesting that cells are capable of sorting miRNA into EVs (40). In support of this a number of sorting mechanisms have been reported and were recently discussed in detail in a review by Groot et al. (41). These mechanisms broadly include RNA-binding proteins such as hnRNPA2B1, membranous proteins involved in EV biogenesis such as Caveolin-1 and posttranscriptional RNA modifications such as 3’-end uridylation (42, 43). Furthermore there is evidence that the miRNA cargo of EVs can be taken up and alter gene expression and biological processes, such as immune response in the recipient cells (44, 45). The intercellular transfer of EV miRNA has therefore been implicated in mediating a range of pathophysiological processes including in the development of COPD (46).

Given the ability of EVs to transfer cargo it is possible they allow distal communication to contribute to or protect against pathological mechanisms underpinning the development of multiple chronic diseases. The following sections will discuss the current research around the potential role of EVs and EV-associated miRNA as a shared disease mechanism that could contribute to the development of COPD and multimorbidity.

## Ageing, Inflammation and the Development of Chronic Diseases

The world's ageing population presents a major challenge for health care services globally, particularly as the prevalence of many chronic diseases increases with age (47, 48). There is accumulating evidence that ageing induces a state of chronic inflammation, that may simultaneously contribute to the development of multiple age-related chronic diseases such as COPD, CVD and osteoporosis (10, 49, 50). This process, known as inflammaging, is characterised by significantly higher levels of circulating pro-inflammatory markers, such as interleukin (IL)-1, IL-6, IL-8, IL-13, IL-18, C-reactive protein (CRP), transforming growth factor-β (TGFβ) and tumour necrosis factor (TNF) (49). Inflammaging is thought to be a consequence of the accumulation of cellular damage due to mitochondrial dysfunction, defective autophagy/mitophagy, an impaired ubiquitin/proteasome system and endoplasmic reticulum stress in combination with exhaustion of endogenous damage-associated molecular pattern (DAMP) clearance mechanism (51–54). Cellular senescence, defined as irreversible cell cycle arrest, has also been suggested to contribute to inflammaging. This process is driven by a variety of mechanisms including the DNA damage response and age-related telomere attrition that activate the senescence-associated secretory phenotype (SASP) wherein cells release high levels of pro-inflammatory factors (55). The number of senescent cells increases with age, generating low-grade inflammation which has widely been implicated in the pathogenesis of age-related diseases (56). Inflammaging is also associated with changes in immune cell function and subset composition with age, known as immunosenescence. The key changes occurring in immunosenescence were recently summarised by Feehan et al. and broadly include reduced phagocytosis, altered immune-modulatory cytokine expression, increased autoimmunity and diminished activation of the adaptive immune response (57). Furthermore there is increasing evidence that COPD and its coexistent conditions represent an acceleration of the ageing process (58, 59).

### EVs as Immunomodulatory Factors in Ageing

Age related changes in the concentration of circulating EVs has been a topic of debate as it remains difficult to accurately measure the concentration of EVs due to the complexity of isolating pure EV populations, detecting EVs and enumerating them (60). Using nano-particle tracking analysis (NTA), Eitan et al. reported that the concentration of circulating EVs decreased with age in human plasma (61). These authors suggested this decrease was partially due to enhanced internalization by B cells, as determined by a FACS-based assay using fluorescently labelled EVs (61). It was also suggested that the reduction in EV concentration with age could be a consequence of an impaired clearance mechanism, given EVs may function to dispose of unwanted proteins and other molecules (61). Later studies using NTA have reported no significant difference in the concentration of plasma EVs from non-smokers, smokers and patients with COPD (62, 63). However using NTA to determine EV concentration has major limitations given it cannot differentiate other particles such as lipoproteins that are commonly co-isolated with EVs. Other studies have demonstrated changes in EV concentration based on common EV markers. For example, the concentration of CD9 positive EVs was shown to be significantly elevated in COPD patients and to correlate with physiological markers of multimorbidity including CRP and IL-6 (64, 65).

Inflammation is a major hall-mark of aging and age related diseases. A number of studies have suggested an age-related increase of EV miRNAs with an anti-inflammatory role, which may act as a compensatory mechanism to oppose the hyperinflammatory state that increases during normal aging and is accelerated in progression of aging-related diseases such as COPD. Plasma EVs from aged mice have been shown to be enriched in miR-192 that functions to suppress the inflammatory response in macrophages (66). This was demonstrated by a significant reduction of IL-6 and IL-1β expression in stimulated murine macrophages following treatment with EVs isolated from miR-192 transfected RAW264.7 cells. Furthermore, exogenous intravenous administration of EVs, isolated from miR-192 transfected RAW264.7 cells, were shown to significantly reduce macrophage recruitment and expression of IL-6, IL-12, TNF, interferon (IFN) and CCL2 in lung tissue of mice following inoculation with inactivated influenza whole virus particles (WVP). Increased levels of miR-192 have also been reported in plasma derived EVs from COPD patients suggesting that the EV miR-192 response to hyperinflammatory state is exaggerated in COPD patents (63). Furthermore, EVs containing miR-192 have been shown to delay diabetic retinopathy and inflammatory responses in rheumatoid arthritis, supporting the role of miR-192 in mediating multiple disease pathways (67, 68). Other miRNA including miR-21, miR-146a and miR-223 have been identified to be elevated in EVs isolated from the plasma of old mice (69). These miRNA were shown to contribute to an anti-inflammatory phenotype, as demonstrated by increased expression of *Arg1*, *Il10* and *Mrc1* in LPS-stimulated macrophages and reduced endothelial cell response to VEGF (69). Similarly to miR-192, studies have shown miR-21, miR-146a and miR-223 to be increased in EVs isolated from patients with COPD or other chronic diseases, such as osteoporosis, CVD and diabetes (70–77). Therefore, these EV miRNA may provide a common link and potential biomarker for accelerated ageing and age-related diseases. However, although these studies suggest an increase in anti-inflammatory EV miRNA as a mechanism to decrease inflammation, other studies have suggested that removal of miRNA, such as miR-223, from alveolar macrophages and T cells *via* EVs releases suppression of the NLRP3 inflammasome thereby promoting activation (78, 79). Further studies using clinically relevant samples are required to establish if the increase in anti-inflammatory EV miRNA observed in ageing and age-related chronic diseases is a mechanism to counteract chronic inflammation or is a mechanism to release suppression of cellular immune and inflammatory pathways.

Mesenchymal stem cells (MSC) have been of particular scientific interest because of their potent immunomodulatory and anti-inflammatory properties. However stem cell exhaustion has been implicated in ageing and several chronic diseases including COPD (80). Huang et al. demonstrated that human MSC EVs from a young donor suppressed the activation of IL-6, IL-1β and TNF in the lung following LPS insult when injected into mice (81). On the other hand, this anti-inflammatory response was not observed for MSC EVs isolated from an old donor. Significantly higher levels of anti-inflammatory miR-223 and lower levels of pro-inflammatory miR-127 and miR-125b were observed in MSC-EVs from young vs old mice which may explain this phenomenon (81). In a separate study, MSC-EVs that originated from older rats were demonstrated to have a lower content of miR-133b-3p and miR-294, two miRNAs that inhibit TGF-β1-mediated epithelial-to-mesenchymal transition, which contributes to fibrosis (82). Reduced expression of miR-133b has also been identified in COPD patients, coronary artery disease and osteoporosis with a range of suggested functions including regulating vascular smooth muscle cells and osteoblast differentiation (83–85). These studies suggest EVs from aged MSC may be less capable of protecting against chronic inflammation and tissue damage, which in turn may promote accelerated ageing and the development of chronic diseases such as COPD.

Endothelial cells that line the lumen of blood vessels not only act as a physical barrier but play a pivotal role in regulating blood flow and immune cell recruitment (86). Ageing and exposure to noxious stimuli induces senescence of endothelial cells which contributes to endothelial dysfunction in both COPD and CVD (87, 88). Epigenetic regulation of DNA damage and senescence has been reported as a pathogenic mechanism linked to endothelial dysfunction in COPD patients (89). Mensà et al. demonstrated that EVs isolated from an *in vitro* model of endothelial replicative senescence enhanced the senescent associated profile in recipient endothelial cells including increased expression of cell cycle inhibitor p16 and SASP factors including IL-6 and IL-8 (90). The mechanism by which EVs from senescent cells can spread premature senescence was suggested to be due to inhibition of the epigenetic regulators DNMT1 and SIRT1 *via* increased levels of EV miR-21 and miR-217 (90). Furthermore, EVs from the plasma of elderly humans and senescent cultured endothelial cells, have been shown to promote calcification in vascular smooth muscle cells, a risk factor for the development of CVD (91). In addition, EVs released from senescent endothelial cells have been shown to be enriched in miR-31 that inhibits osteogenic differentiation of MSCs, providing a possible link between endothelial dysfunction and osteoporosis (92).

Research over recent years into characterising the age-related changes in EVs reveals diverse functional changes in EVs with age and suggest it is unlikely to be as simple as concluding they either promote or inhibit “inflammaging” and chronic disease. More likely, there are different subtypes of EVs with different functions depending on their origin and the mechanism by which they are released. Further research is required to characterise these distinct EV populations across the human lifespan. Increased susceptibility to infection and the multimorbidity observed in COPD may be partially attributed to the reduced immune function observed with ageing as discussed in the following section.

## Respiratory Infections, Inflammation and the Development of Chronic Disease

Respiratory infections are the primary driver of COPD exacerbations, which lead to worsening of symptoms and increased risk of hospitalisation and mortality (93–96). Respiratory viruses, including rhinovirus, respiratory syncytial virus (RSV) and influenza, are commonly associated with COPD exacerbations whilst colonisation of bacteria such as non-typeable *Haemophilus influenzae* (NTHi), *Streptococcus pneumoniae* and *Moraxella catarrhalis* in the airways of COPD patients is common during both stable disease and exacerbations (12, 13, 97–100). In particular, acquisition of new bacterial strains appears to be associated with an increased risk of COPD exacerbations (101, 102). Additionally, acute childhood infections are thought to play a role in initiating pathological mechanisms which could predispose individuals to chronic diseases in later life (103, 104). The potential long-term impact of respiratory infections has been exemplified by the recent SARS-CoV-2 pandemic with severe COVID-19 leading to multiple organ damage and a range of long-term systemic effects (105, 106). In addition, greater morbidity and increased mortality has been observed following infection with SARS-CoV-2 in individuals with existing chronic diseases such as COPD, CVD and diabetes (107–111). However, the long-term impact of COVID-19 disease on the body and resultant pathological mechanisms that could drive susceptibility to lung diseases, such as COPD and other systemic diseases, is still unravelling.

### Immune and Inflammatory Role of EVs in Response to Respiratory Infection

Respiratory infections have been reported to trigger increased levels of lung-derived EVs released from alveolar macrophages and epithelial cells (112, 113). EVs released in response to respiratory infections, that are frequently detected in AECOPD, contribute to the production of immune and inflammatory mediators. A recent study reported significant differences in small RNA EV cargo released by the human alveolar epithelial A549 cell line when infected with RSV (114). These EVs were shown to activate the innate immune response as demonstrated by increased production of cytokines and chemokines including CXCL10, CCL5 and TNF in other A549 cells and human monocytes (114). On the other hand, proteomic characterisation of EVs released from human macrophages upon influenza infection revealed EVs may directly transfer pro-inflammatory cytokines (115). EVs have also been shown to upregulate type I IFNs, a key mediator of anti-viral responses. Liu et al. demonstrated that EVs produced by influenza infected A549 cells induced IFN production to inhibit viral replication through upregulation of miR-1975 (116). Similarly, BALF EVs from a murine model of highly pathogenic avian influenza have been reported to have an increased level of miR-483-3p and to potentiate IFN immune response (117). Furthermore, pattern-associated molecular patterns (PAMPs)-containing EVs have also been shown to stimulate a proinflammatory response in macrophages through TLR and enhance T cell activation (118, 119). In addition SARS-CoV-2 viral RNA within EVs derived from A549 epithelial cells has been shown to be internalised by cardiomyocytes and up-regulate inflammatory genes (120). The systemic dissemination of inflammation by macrophage-derived EVs has also been demonstrated using a LPS challenged murine model (121). This effect was proposed to be due to the interaction of histones on the outer surface of vesicles with TLR4 (121).

Endothelial dysfunction driven by respiratory infections has been associated with higher incidence of acute cardiovascular events following COPD exacerbations (122, 123). The concentration of endothelial EVs has been reported to be elevated in patients with COPD who have frequent exacerbations and may contribute to systemic effects by mediating coagulation, vascular tone and angiogenesis (124). Indeed coagulant proteins have been reported to be enriched in EVs in the plasma of COPD and cardiovascular patients (62, 125). EVs released in response to respiratory infections may be a contributing mechanism. For example, SARS-CoV-2 infection induces the release of tissue factor (TF) positive EVs into the circulation that was suggested to contribute to thrombosis and mortality in patients with COVID-19 (126–129).

Although there are a growing number of studies that report that EVs released in response to infection are pro-inflammatory and can induce systemic inflammation, there is currently no direct research into whether host EVs released in response to infection contribute to development and exacerbation of chronic diseases including COPD. However plasma EVs from COPD patients have been shown to contain higher levels of miR-125b that has previously been suggested to directly reduce antiviral signaling and cause exaggerated inflammation and impaired antiviral responses to IAV (63, 130). Therefore, further research is required to determine if dissemination and uptake of EVs contribute to the exacerbated or persistent inflammation observed in chronic disease in response to infection. In addition, given that EVs have been shown to modulate the IFN response, they may have useful anti-viral therapeutic applications. The potential of IFN treatment in the prevention of virally induced exacerbations in COPD is currently being investigated (131, 132).

Gram-negative bacteria associated with COPD, such as NTHi and *M. catarrhalis*, have also been shown to release EVs known as outer membrane vesicles (OMVs). OMVs are a similar size to host EVs, approximately 20 to 350 nm and can also export a range of cargo including proteins and small RNAs (133). Despite a wide number of studies demonstrating the effects of OMVs in host-microbe interactions, including their ability to enter the systemic circulation and induce a variety of immunological and metabolic responses, the exact mechanisms of bacterial vesicles and their content are still largely unknown (134, 135). Bacterial OMVs can deliver their cargo to a range of host cells including epithelial cells, neutrophils and macrophages and subsequently stimulate an inflammatory response (133, 136, 137). For example, OMVs of *M. catarrhalis* can bind to TLR2 on epithelial cells and are subsequently internalized, causing a pro-inflammatory response and increased levels of IL-8 (138). Furthermore, OMVs have been implicated in the formation of biofilms that increase tolerance to antimicrobial treatments and the immune system (99, 139). While the presence of NTHi and *M. catarrhalis* have been associated with a heightened risk of COPD exacerbation, the mechanisms for this remains unclear. Given that NTHi and *M. catarrhalis* have been shown to release OMVs, that can activate host immune responses as well as potentially support bacterial colonization, they may provide a novel mechanism contributing to the nature of chronic and recurrent bacterial infections in AECOPD and systemic disease. In support of this, a recent review highlighted the role of *Porphyromonas gingivalis* in promoting the development of related systemic diseases including diabetes and cardiovascular disease through long distance transmission of OMVs (140, 141). Further research is, therefore, required to determine role of OMVs in COPD and other systemic diseases.

Our understanding of the role of EVs in activating the host immune response against respiratory pathogens, or facilitating infection, remains sparse. The release of pro-inflammatory EVs in response to infection may have the ability to induce inflammation, both locally in the lung and distally, contributing to the pathology of COPD and allied chronic diseases. Further studies are required to elucidate the mechanisms by which EV cargoes modulate the immune response of recipient cells over the course of infection and whether this is dysregulated in chronic diseases and could contribute to an impaired immune response. In contrast, other environmental risk factors such as CS have been shown to increase the incidence of multiple chronic diseases.

## Respiratory Toxins, Inflammation and the Development of Chronic Disease

Exposure to respiratory toxins, such as CS and air pollution, has been shown to be the primary risk factor for COPD as well as significantly increasing the risk of developing CVD and diabetes complications. Sustained exposure to harmful stimuli results in profound functional and structural changes to the airway epithelium including changes in mucous production, impairment of epithelium regeneration and reduction in cilia development (142). In addition, smoking contributes to immune dysregulation including an increase in pro-inflammatory effects such as increased immune cell recruitment as well as immunosuppressive effects including suppression of immune cell effector functions (143). Furthermore, the damage caused by respiratory toxins has been associated with accelerated ageing and increased susceptibility to infections that contribute to chronic disease pathology, as discussed previously. Studies in smokers with mild-moderate COPD have shown that the relationship between COPD and CVD is mediated through established cardiovascular risk factors such as tobacco smoking rather than through COPD itself (18). As with respiratory infections, a link between CS exposure in early life and the development of chronic disease during adulthood has also been demonstrated (144). However, while smoking cessation slows the rate of decline of pulmonary function in COPD patients it does not halt disease progression and systemic inflammation persists. Therefore, EVs may provide a mechanism contributing to the self-perpetuating spread of systemic inflammation.

### Immune Modulating EVs Released in Response to Respiratory Toxins

Oxidative stress induced by the imbalance between oxidants and antioxidants from exposure to CS has been shown to increase the levels of EVs released by the airway epithelium. Benedikter et al. proposed that exposure of the BEAS-2B human bronchial epithelial cell line to cigarette smoke extract (CSE) increases the release of EVs due to oxidative depletion of surface thiols (145). In support of this proposal, antioxidants such as N-acetyl-L-cysteine (NAC) were shown to prevent CSE-associated increases in EVs (145). However, it is possible that the increased levels of EVs detected were due to reduced EV uptake, as another study reported reduced EV uptake following exposure to CSE that was reversed by the presence of NAC (146). While there is overwhelming evidence that oxidative stress and oxidative damage play a pivotal role in the pathogenesis of COPD and other systemic diseases, further research is required to determine if redox-dependent thiol modification is a potential mechanism contributing to the modulation of EVs in multimorbid patients’ (147).

Exposure to respiratory toxins upregulates inflammatory pathways that result in increased immune cell recruitment and release of pro-inflammatory mediators commonly upregulated in COPD and other chronic diseases. EVs released in response to respiratory toxins have been shown to promote cytokine release in epithelial cells. Heliot et al. reported that EVs isolated from the BAL of smokers increased secretion of IL-6 by BEAS-2B cells (148). Similarly, Martin et al. reported that EVs released by THP-1 macrophages which were exposed to PM_2.5_ promoted the release of IL-6 and TNF from BEAS-2B cells (149). The release of EVs from airway epithelial cells in response to respiratory toxins has been reported to induce recruitment of monocytes and activation of macrophages (30, 150). One study reported that EVs released from BEAS-2B cells under oxidative stress activate macrophages and promote expression of TNF, IL-1β, and IL-6 though increased EV levels of miR-320a and miR-221 (151). Increased levels of miR-221 have been reported to enhance smoking-induced inflammation in COPD (152). Furthermore, increased EV miR-320a has been reported in the plasma of osteoporosis patients and was suggested to impair osteoblast function and induces oxidative stress (77, 153). The manifestation of systemic disease has been suggested to be potentially caused by elevated levels of pro-inflammatory Wnt5a in circulating EVs in response to smoking and in COPD patients (154). Elevated levels of Wnt5a have also been demonstrated in other chronic diseases including heart failure (155). In addition, the production of circulating procoagulant EVs in response to CS has been demonstrated, further supporting the role of EVs as a mechanism contributing to the systemic effects of inhalation of noxious stimuli (156). Overall these studies suggest that EVs released in response to noxious stimuli contain cargo that have been associated with chronic disease and promote inflammation.

In COPD, airway epithelial cells and immune cells secrete an increased level of proteolytic enzymes resulting in chronic inflammation and destruction of lung parenchyma (7, 157, 158). Li et al. reported a 3-fold increase in EV matrix metalloproteinase (MMP)-14 released from macrophages following exposure to CSE (159). In addition, EVs have been found to be associated with neutrophil elastase that was suggested to contribute to the ability of EVs to degrade extracellular matrix and promote alveolar destruction (160). These studies provide a mechanism by which EVs, released in response to noxious stimuli, may contribute towards tissue damage that promotes airway remodelling. EVs have also been reported to play a role in airway remodelling by mediating fibrosis through epithelial-mesenchymal transition (EMT) and myofibroblast differentiation. CSE has been shown to increase miR-210 and miR-21 in human bronchial epithelial cell EVs, leading to suppression of autophagy and an increase in myofibroblast differentiation (161, 162). On the other hand, He et al. reported a reduction in EV miR-21 released from CSE-treated BEAS-2B cells, which indirectly modulated EMT by alleviating the polarization of M2 macrophages (73). Furthermore, Coresello et al. reported no significant changes in the level of EV miR-21 released from human small airway epithelial cells in response to CSE (163). Variations in the type of airway epithelial cell models, CS sources and EV isolation methods could account for the differences in EV miR-21 levels reported between the studies (164). As mentioned previously, miR-21 has been identified in elevated levels in EVs in response to risk factors such as ageing, as well as from patients with chronic disease including COPD, CVD, diabetes and oseteoporosis. Furthermore, miR-21 has been widely reported as an inflammatory mediator and suggested as a key switch in the inflammatory response (165). Therefore it will be vital to complete further research with standardised EV isolation and characterisation techniques to fully understand the biologically relevant effects of EV miR-21 and its contribution to multimorbidity.

Exposure to respiratory toxins damages the epithelial and endothelial barrier, This contributes to the “overspill” of inflammatory mediators, including EVs produced in the lung, to the circulation. An increase in circulating EVs and alteration of their cargo in response to noxious stimuli may contribute to systemic inflammation and the development of chronic diseases such as COPD. Further studies are required to understand if a distinct EV population can be used to discern the smokers who will go on to develop multiple chronic diseases.

## Future Perspectives

**Figure 3:**
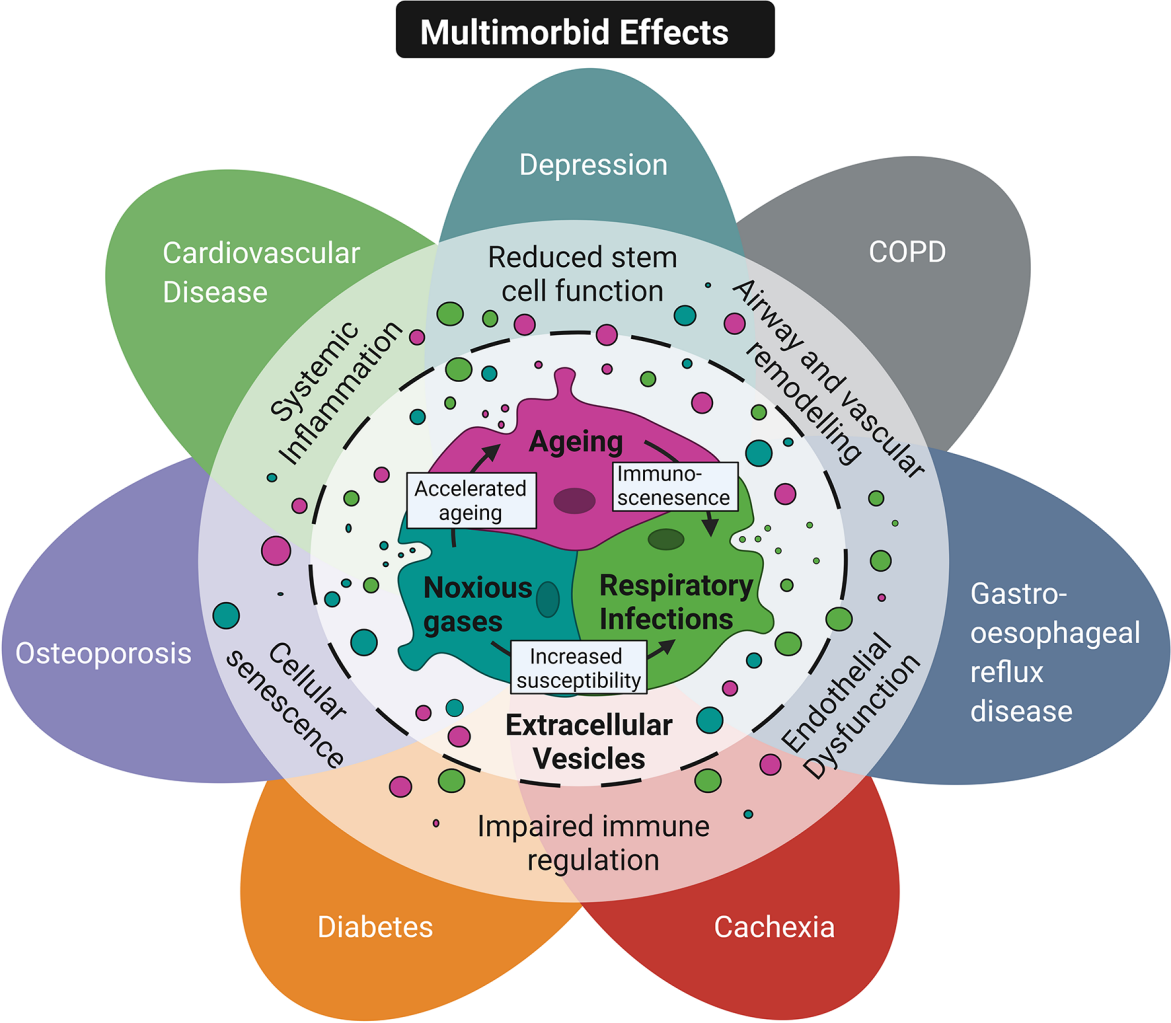
Conceptual diagram of EVs as a common mechanism in multimorbidity. Shared risk factors including ageing, inhalation of noxious stimuli and respiratory infections are thought to contribute to multimorbidity in COPD patients. These risk factors can exacerbate each other. For example ageing promotes immunosenescence and reduces the ability of the immune response to neutralise infection. In addition, smoking increases susceptibility to infection and accelerates features of ageing. These risk factors have also been shown to promote the release of EVs that mediate key pathological features in multimorbid COPD patients' such as reduced stem cell function, airway and vascular remodeling, endothelial dysfunction, impaired immune regulation, cellular senescence and systemic inflammation. Created with BioRender.com.

The field of EV research is still relatively new and our understanding of the role of EVs is rapidly evolving. Given the proposed function of EVs in mediating pathways that are central to multiple chronic diseases they may pose as novel biomarkers or useful therapeutic targets (**Figure 3**). However, no definitive link has yet been established between circulating EVs and the development or exacerbation of chronic diseases. A critical question is whether EVs directly contribute to associated pathology or are simply a consequence of the disease. The impact of exposure to different risk factors in modulating disease relevant EVs needs to be determined, alongside their relative contribution and combinatory effects. Furthermore, while a number of studies have alluded to EVs as systemic immune modulators able to reach distant organs, further studies are required to validate this theory.

Current studies investigating the role of EVs in chronic diseases have been limited by the lack of a gold standard for EV isolation and functional characterisation. EV profile characterisation has been demonstrated to be dependent on the experimental models and techniques applied, with variable particle yield, genomic and proteomic EV profiles reported between different methodologies (25). Application of recently emerging technologies will allow better isolation and characterisation of distinct EV populations. For example, microfluidic technologies, such as asymmetric flow field-flow fractionation (AF4), have recently been demonstrated as an improved technique for isolating distinct nanoparticle subpopulations and therefore allow better characterisation of heterogeneous EV populations (166). Furthermore, there is growing interest in techniques that allow single-EV analysis and therefore can tease apart the distinct biophysical and molecular properties of individual EVs in a heterogeneous population. One recent example is single-particle interferometric reflectance imaging (SP-IRI), recently developed and sold as an automated platform called the ExoView system (NanoView Biosciences) (167, 168). This system can be used for multiplexed analysis and allows simultaneous sizing and protein profiling. Once techniques for EV isolation and characterisation are standardised, the use of multi-omics approaches, including transcriptomic, proteomic, metabolomic, and lipidomic would be beneficial to better understand the function and relationship of EV biomolecules (169). However, this will require more transparent reporting of methodological details and increased data availability in future studies.

Another issue is that our current understanding of the immunomodulatory role of EVs in response to biological and environmental risk factors is based largely on *in vitro* cell culture models that may oversimplify the *in vivo* functions of EVs due to the limited intercellular interactions. Moreover, many *in vivo* studies into the function of EVs have been based on murine studies that have previously shown to have limitations regarding recapitulation in the human biological system. Therefore, further work using relevant human *ex vivo* and co-culture models are required to obtain clinically relevant data. Additionally, while clinical samples provide the biological complexity necessary, they should include a well characterised patient cohort which is large enough to reduce bias due to patient heterogeneity. In clinical studies, analysing EVs across a range of chronic diseases using a standardised isolation protocol and with stratification of multimorbid patients will also be necessary to compare disease related changes in EV cargo.

## Conclusion

On review of recent EV literature it is apparent that there is overlap in EV cargo shown to be altered across a range of chronic diseases and in response to disease risk factors such as ageing, infection and smoking. Furthermore many of these changes in EV cargo mediate pathological features, such as chronic inflammation, that are central to multimorbidity in COPD patients and therefore may provide novel diagnostics and therapeutics. However, further studies investigating the function of EVs in multimorbidity using physiologically relevant, disease specific, *ex vivo* models are required.

## Author Contributions

LR: conceptualization, investigation, literature searching, analysis, project administration, writing original draft, reviewing and editing. CS: supervision, conceptualization, reviewing & editing. AW: supervision, reviewing & editing. KS: supervision, conceptualization, reviewing & editing. TW: supervision, conceptualization, reviewing & editing. All authors contributed to the article and approved the submitted version.

## Funding

This work was funded by an MRC Integrated PhD studentship awarded for LR’s doctoral studies.

## Conflict of Interest

KS reports grants from AstraZeneca, outside the conduct of the study. TW reports grants and personal fees from AstraZeneca, outside the conduct of the study; personal fees and other from MMH, grants and personal fees from GSK, grants and personal fees from AZ, personal fees from BI, grants and personal fees from Synairgen, outside the submitted work.

The remaining authors declare that the research was conducted in the absence of any commercial or financial relationships that could be construed as a potential conflict of interest.

## Publisher’s Note

All claims expressed in this article are solely those of the authors and do not necessarily represent those of their affiliated organizations, or those of the publisher, the editors and the reviewers. Any product that may be evaluated in this article, or claim that may be made by its manufacturer, is not guaranteed or endorsed by the publisher.
